# Epithelial Mesenchymal Transition: A New Insight into the Detection of Circulating Tumor Cells

**DOI:** 10.5402/2012/382010

**Published:** 2012-04-11

**Authors:** Guislaine Barrière, Michel Tartary, Michel Rigaud

**Affiliations:** Clinical Laboratory, Astralab Department of Specialized Analyses, 87000 Limoges, France

## Abstract

Many research groups reported on the relation between circulating tumor cells (CTCs) in peripheral blood and worse prognosis for metastatic cancer patients. These results are based on CTCs counting and did not take into account molecular characteristics of cells. To establish CTCs as a reliable and accurate biological marker, new technologies must be focused on CTC subpopulations: dedifferentiated circulating tumor cells (ddCTCs) arising from epithelial mesenchymal transition (EMT). To select and detect them, different methods have been proposed but none has still reached the goal. Technical progress and translational research are expected to establish CTCs as a real marker. Thus CTC evaluation profiling for each patient will lead to personalize followup and therapy.

## 1. Introduction

Human cells can be classified as either epithelial or mesenchymal and are molecularly characterized by specific expression of genes. In early embryonic morphogenesis, epithelial cells give rise to mesenchymal cells by a reversible reaction, epithelial mesenchymal transition (EMT), between the two phenotypes. Analogous cell status modification is observed in human cancer cells [1–3]. Numerous studies have established a link between EMT markers in primary tumor cells and aggressive clinical behaviour [4, 5]. Spread of epithelial tumors to an anatomically distant site seems to occur almost totally via the process of hematogeneous dissemination [6, 7]. Moreover, the initiation of metastasis may be an early event in tumor biology. Circulating tumor cells (CTCs) have been postulated to be critical to this process. In 1869, Ashworth discovered analogous cells to those of a primary tumour in the postmortem patient's blood and named them Circulating Tumor Cells [8]. Today, the term CTC encompasses all types of cells, which are considered as foreign entities in the blood having some cancerous characters. Evidence has emerged that CTCs present a heterogeneity as the one described for primary tumor cells. Among CTC subpopulations, cancer stem and mesenchymal cells have to be taken into account. We proposed to name these cells dedifferentiated circulating tumor cells (ddCTCs).

CTC analysis is generally based on numeration, which is considered to have a prognostic value, and the CellSearch system (Veridex corporation, USA) has been cleared by FDA as an aid to monitor patients with metastatic breast, prostate, and colon cancer [17–19]. The surface epithelial cell adhesion molecule (EpCAM) identifies epithelial cells circulating within the blood. Cells lacking CD45 but expressing cytokeratins (CK8, CK18, and CK19) are identified as CTCs. This analytical method is able to predict free disease and overall survival but cells arising from EMT often escape detection [20]. Apart from counting, molecular characterization of CTCs is of interest for larger purposes. Other methods better isolate cell subpopulations having key roles in metastatic process. We focus our review on these analytical developments and their use for an accurate characterization of ddCTCs.

## 2. Epithelial Mesenchymal Transition

EMT is a morphogenetic process in which cells lose their epithelial characteristics and gain mesenchymal properties during embryogenesis, wound healing, and carcinoma invasion and acquire a fibroblast-like morphology. These mesenchymal cells may revert to an epithelial phenotype allowing growth of metastasis [21]. It remains to be proved that all the genes involved in physiological EMT pathways are implemented in carcinoma cells migration. Vazquez-Martin et al. demonstrated that EMT status rather than EMT functioning establishes stemness [22]. They indicated that overexpression of some EMT regulators may be sufficient to efficiently generate the breast cancer stem cell phenotype and their inhibition reduces mammospheres formed by MDA-MD-231 cells growing in sphere medium. Thus, aberrant disrupted signaling pathways sustain cancer cell division and tumor growth. Thereby their results suggest that epithelial cancer cells go astray to acquire motility, therapeutic resistance, and stemness characters without driving a complete EMT program. This could explain the diversity of CTC subpopulations.

 Some cells leave the primary tumor after EMT, migrate separately, and look like mesenchymal cells. To borrow such a way, there are modifications of protein structures implicated in cell junctions: tight junctions, adherens junctions, desmosomes, and gap junctions. The latter kept cells together, unlike to mesenchymal cells that interact with their neighbours only at focal contact without forming layers. Other migrating cells can have unsteady phenotype, which corresponds to a partial EMT characterized by maintenance of cell-cell adhesion structures that do not preclude active cell migration. Therefore, new detection methods must take into account the diversity of circulating cell subsets and relevant markers have to be developed [23]. Until now, quality of CTC analyses has been biased by currently used enrichment methods. Protocols often rely on marker expressions (EpCAM, cytokeratins) possibly leading to a loss of less differentiated tumor cells. Cells obtained by EpCAM enrichment are heterogeneous and those not expressing epithelial markers can be detected by using EMT-like characteristics [24]. Moreover, some cells lack EpCAM expression and are missed when CTCs are captured with solely EpCAM-based technology [25]. Owing to this subpopulation diversity, use of more than one antibody is a necessity.

## 3. Mesenchymal and Cancer Stem Cells

Ontogeny of cancer stem cell-like has not been fully delineated. EMT status mediates invasive chemo/radioresistance properties and mesenchymal morphology of cancer cells. It endows cells with stemness features. These dedifferentiated cells were initially described in breast cancer by Al-Hajj et al. [26]. Their hallmark is CD44^+^/CD24^−/low^ immunophenotype. However, Meyer et al. demonstrated that such cells can give rise to CD44^+^/CD24^+^ populations with tumor initiating potential [27]. Some authors noticed that the stemness character of cancer cells is detected by ALDH1 expression [28]. These results underline the difficulty to classify the subsets of dedifferentiated cells. Numerous studies described epithelial tumors as a mixture of cell clones, and so, such an heterogeneity must be found among CTCs.

EMT is promoted by several signaling pathways: TGF*β*, Wnt, Notch, hedgehog, and growth factors [29–34]. They converge towards induction of transcriptional factors (Snail1, Slug, ZEB1 ZEB2, E47, and Twist), which suppress E-cadherin expression [35, 36]. In epithelial cells, *β*-catenin colocalizes with E-cadherin at cytoplasmic membrane and enables the link between adherens junctions and actin cytoskeleton. When E-cadherin is lowered, *β*-catenin translocates to nucleus and participates to transcriptional regulation of EMT. In EMT regulation, *β*-catenin plays a central role. When E-cadherin is downloaded, cells express N-cadherin, a protein that is associated to migration invasion and metastasis [37]. Some properties of mesenchymal cells are issued from PI3Kase/Akt pathway, such as resistance to apoptosis and proliferation effects [38, 39]. Isoforms of Akt control EMT and stem cell renewal, regulations depending on the equilibrium between isoforms 1 and 2 rather than on the total Akt. The activity of Akt is negatively regulated by PTEN (Phosphatase and Tensin Homologue). PTEN/PI3Kase/Akt pathway seems to be a promising target in the treatment of breast cancer patients [40, 41]. A special focus has to be made on Twist and BMI1. These two proteins are essential in induction, maintenance of EMT, and acquisition of stemness by mesenchymal cells (BMI1 is a polycomb group protein that maintains self-renewal). Yang et al. suggested that Twist1 and BMI1 cooperatively act to promote cancer dedifferentiation and metastasis [42]. They demonstrated that BMI1 suppression upregulates p16INK4a expression and reduces CD44^+^, ALDH^+^, and side population cells leading to decrease tumorosphere formation.

MicroRNAs (small 19–22 nucleotides long noncoding RNA) that inhibit gene expression at the posttranscriptional level are implicated in EMT and stemness. Among them, mir200c seems to be a pivotal one [43, 44]. ZEB1 and ZEB2, EMT mediators, are targeted by mir200c. Moreover, it negatively regulates BMI1 involved in self-renewal of stem cells. Another microRNA, like let-7, plays major roles in invasion metastasis and dedifferentiation of cancer cells [45].

This burst of research and results concerning mesenchymal and cancer stem cells essentially devoted to primary tumors and cancer cell lines led numerous laboratories to start a translational research on this type of cells in the blood.

## 4. Comparative Analyses of CTC Subpopulations

New described methods propose molecular characterization of cells. They differ from those limited to numeration, which only bring information about the prognosis. They would be able to establish an accurate assessment of the residual disease and to develop personalized therapy. The followup of these ddCTCs in the blood might be essential to test efficacy in novel drug trials. In this context, we discuss major recent publications taking into account the EMT aspect for CTC analysis, even if they only studied a few number of markers. Quoted data are summarized in Table 1.

Aktas et al. described that tumor cells spreading from the primary tumor into the circulation may undergo phenotypic changes known as EMT [9]. They evaluated 226 blood samples of 39 metastatic breast cancer (MBC) patients during the followup of palliative chemo-, antibody-, or hormonal-, therapy. The test required the enrichment of CTCs from blood by using AdnaGen technology (AdnaGen AG, Langenhagen, Germany) with a special washing procedure. A singleplex PCR assay for ALDH1 and a multiplex PCR for EMT markers (Twist1, Akt2, PI3K*α*) were used. They showed that EMT characteristics are detectable in some CTCs analyzed in MBC samples, suggesting a negative prognostic impact as EMT switch leads to decreased apoptosis and to the development of chemoresistance. Furthermore, overexpression of ALDH1 in substantial number of their samples demonstrated that CTCs often display stemness. Their results underlined the diversity of subpopulations but the classification of CTC patterns seems not very clear.

Theodoropoulos et al. assessed the expression of CD44, CD24, and ALDH1 on cytokeratin positive CTC of 30 patients with MBC using triple-marker immunofluorescence combined with confocal microscopy [10]. They reported the presence of CD44^+^/CD24^−/low^ and ALDH1^high^/CD24^low^ CTCs. Their cytological method enables characterization of individual CTC and seems to be specific and sensitive; nevertheless, this research approach is not feasible for daily practice.

The high frequency of CTC coexpressing epithelial, mesenchymal, and stem cells markers has been demonstrated by Armstrong et al. [11]. CTCs were processed by using the CellSearch EpCAM-based immunocapture method, and expression of some epithelial, mesenchymal, and stemness markers was studied. In patients with metastatic castration-resistant prostate cancer, Epcam, cytokeratins, and E-cadherin were simultaneously expressed with vimentin, N-cadherin, O-cadherin, and a stem cell marker CD133. They equally found coexpression of cytokeratins, vimentin, and N-cadherin in CTCs of MBC. Thus they demonstrated that CTCs are solely not a population but exist in different transitional states from epithelial to stem cells.

The expression of Twist and vimentin in CTCs of patients with early breast cancer or MBC was studied by Kallergi et al. [12]. This investigation was done by double immunofluorescence on isolated peripheral blood mononuclear cell cytospins using anti-CK anti-Twist or anti-vimentin antibodies. Triple staining revealed that all CK^+^/Twist^+^ or CK^+^/vimentin^+^ cells were also CD45^−^. As others, these authors demonstrated that both mesenchymal and epithelial markers can be coexpressed in a same cell. The particularity of their experiment lies on the use of negative selection procedure for CTC isolation. Before cytospin, blood is incubated with Dynal CELLection coated with anti-CD45 antibody. Twist and vimentin markers, suggesting EMT, were identified more often in patients with metastatic disease than in early-stage breast cancer. This fact supports that EMT is involved in the invasive potential of CTCs.

A hybrid (epithelial/mesenchymal) phenotype of CTCs was also demonstrated by Lecharpentier et al. in patients with metastatic non-small-cell lung cancer (NSCLC) [13]. Enrichment of cancer cells was made by blood filtration using ISET methodology. They are triply labelled with fluorescent anti-vimentin, anti-pan-keratin antibodies, and SYTOX orange nuclear dye. This publication showed for the first time the existence of CTCs with an epithelial mesenchymal phenotype in patients with NSCLC.

To test efficacy of neoadjuvant therapy, Mego et al. studied the expression levels of EMT-inducing transcription factors: Twist1, SNAIL1, SLUG, ZEB1, and FOXC2 in CD45-negative CTCs [14]. They demonstrated that 15% of patients with primary breast cancer overexpressed at least one of the EMT markers and that any of these markers was more likely to be detected for patients who received neoadjuvant therapies than those who did not receive. Thus their results underlined that CTCs with EMT phenotype may occur in the peripheral circulation of patients with primary breast cancer. Although there was no statistical correlation between the overexpression of any EMT transcription factors and the presence of CTCs detected by either CellSearch or AdnaTest, they showed that CTC-positive patients detected by these two methods had a higher tendency to overexpress EMT markers compared to patients with no CTCs. Once again, these results target the heterogeneity of CTC population.

Pecot et al. demonstrated that EMT-derived CTC populations are likely missed by current techniques and proposed to explore new methods beyond the use of EpCAM and CK-based antibody platforms for capturing and detecting previously unrecognized CTC subpopulations [15].

In a recent experiment, we detected ddCTCs in patients with early breast cancer diagnosis [16]. Blood analysis for 61 patients has been performed before any therapy at baseline diagnosis. Three phenotypes were distinguished and characterized by EMT markers (Twist, PI3K*α*, Akt-2), stemness markers (ALDH1, Bmi1, CD44), or a mixture of the two previous ones. AdnaGen method was used for enrichment cell selection, and ddCTCs were characterized by RT-PCR. Among 61 included patients, 21 (39%) were positive for ddCTCs. Statistical results indicated a relationship between axillary node invasion and detection of ddCTCs but the significance level had a borderline value (*P* = 0.05). None of these patients apart one showed epithelial circulating cells. It is the first time that ddCTC presence in the blood is demonstrated with such a high rate at least when the primary breast tumor is diagnosed without metastasis. We demonstrated that EMT, leading to mesenchymal and stemness phenotypes of CTCs, is an early event in the dissemination process.

## 5. Perspectives for CTC Isolation and Characterization

Study of publications dealing with CTC indicates that the lack of standardization of technology hampers the implementation of CTC measurement in clinical routine practice. Some major biological issues are still unresolved. Epithelial cell-surface markers are widely used to trap CTC. Enrichment steps are necessary to increase isolation success rate. In positive selection by immunomagnetic beads, the antibody mostly coated is EpCam. The latter may not be optimal for detecting a heterogeneous population of CTCs including those with a mesenchymal phenotype. To overcome this failure, it seems to us that use of a bead mixture coated with different antibodies is more efficient. Research on specific cancer cell antigens could improve the selectivity of the enrichment step. Moreover, depletion of hematopoietic cells may help to avoid false positive results due to illegitimate expression of mesenchymal or stemness markers [46–49]. The important technical issue is to expand CTC usefulness by increasing the specificity of results leading to establish a real clinical monitoring. In case a combination of specific antibodies against cancer cells can be used, it would allow a better step of enrichment able to capture epithelial, mesenchymal, and cancer stem cells. Thereafter, cell sorting by dielectrophoresis would enable to qualify each single cell at the molecular level [50]. This improvement would conduct to establish a personalized profile for patients. The latter can thus be done by qRTPCR of EMT and/or stemness markers, as now qRTPCR is feasible on a single cell [51]. Such new technologies would demonstrate the potential value of CTCs as markers in the clinical management of cancer.

## 6. Conclusion

The multiplicity of methods described in the literature shows how it is difficult to analyse CTCs and no method has still been endorsed by FDA. To establish CTCs as powerful markers in oncology, optimization and standardization of technologies, based on physics and molecular biology, is essential for precise and reproducible determination. A marker must be an indicator able to measure and evaluate a normal or pathological biological process. If this goal is reached, CTCs would be qualified as a biological marker. A standardized method for CTC analyses would contribute to establish a personalized medicine and to cope with patients in oncology.

## Figures and Tables

**Table 1:** EMT-derived ddCTC. ddCTC patterns from epithelial differentiation to mesenchymal phenotype are summarized by data reported in quoted publications. Epithelial and mesenchymal markers can be coexpressed.

Authors	Cell enrichment and technology	Analysed characteristics of CTC subpopulations		
		Epithelial	Mesenchymal	Stemness
Aktas et al. [9]	AdnaGen and RTPCR Density gradient centrifugation		Twist1, AKT2, PI3Ka	ALDH1 CD44^+^/CD24^−low^
Theodoropoulos et al. [10]	Cytospin, Immunofluorescence microscopy	CK		ALDH1
Armstrong et al. [11]	CellSearch	EpCAM, CK, E cadherin	Vimentin, Ncadherin, O cadherin	CD133
Kallergi et al [12]	Density gradient centrifugation Cytospin and confocal microscopy	CK	Twist1, Vimentin	
Lecharpentier et al. [13]	ISET filtration	panCK	Vimentin	
Mego et al [14]	Depletion of EpCAM and CD45+cells RTPCR		Twist, SNAIL1, SLUG ZEB1, FOXC2	
Pecot et al. [15]	Microchannel platform	CK	Vimentin, Twist Ncadherin, SNAIL	
Barrière et al. [16]	AdnaGen and RTPCR		Twist1, AKT2, PI3Ka	ALDH1 BMI1, CD44

## References

[1] Kalluri R, Weinberg RA (2009). The basics of epithelial-mesenchymal transition. *Journal of Clinical Investigation*.

[2] Klymkowsky MW, Savagner P (2009). Epithelial-mesenchymal transition: a cancer researcher’s conceptual friend and foe. *American Journal of Pathology*.

[3] Tomaskovic-Crook E, Thompson EW, Thiery JP (2009). Epithelial to mesenchymal transition and breast cancer. *Breast Cancer Research*.

[4] Sarrió D, Rodriguez-Pinilla SM, Hardisson D, Cano A, Moreno-Bueno G, Palacios J (2008). Epithelial-mesenchymal transition in breast cancer relates to the basal-like phenotype. *Cancer Research*.

[5] Yang J, Mani SA, Donaher JL (2004). Twist, a master regulator of morphogenesis, plays an essential role in tumor metastasis. *Cell*.

[6] Hanahan D, Weinberg RA (2000). The hallmarks of cancer. *Cell*.

[7] Chambers AF, Groom AC, MacDonald IC (2002). Dissemination and growth of cancer cells in metastatic sites. *Nature Reviews Cancer*.

[8] Ashworth TR (1869). A case of cancer in which cells similar to those in the tumors were seen in the blood after death. *Australian Medicine Journal*.

[9] Aktas B, Tewes M, Fehm T, Hauch S, Kimmig R, Kasimir-Bauer S (2009). Stem cell and epithelial-mesenchymal transition markers are frequently overexpressed in circulating tumor cells of metastatic breast cancer patients. *Breast Cancer Research*.

[10] Theodoropoulos PA, Polioudaki H, Agelaki S (2010). Circulating tumor cells with a putative stem cell phenotype in peripheral blood of patients with breast cancer. *Cancer Letters*.

[11] Armstrong AJ, Marengo MS, Oltean S (2011). Circulating tumor cells from patients with advanced prostate and breast cancer display both epithelial and mesenchymal markers. *Molecular Cancer Research*.

[12] Kallergi G, Papadaki MA, Politaki E, Mavroudis D, Georgoulias V, Agelaki S (2011). Epithelial to mesenchymal transition markers expressed in circulating tumour cells of early and metastatic breast cancer patients. *Breast Cancer Research*.

[13] Lecharpentier A, Vielh P, Perez-Moreno P, Planchard D, Soria JC, Farace F (2011). Detection of circulating tumour cells with a hybrid (epithelial/ mesenchymal) phenotype in patients with metastatic non-small cell lung cancer. *British Journal of Cancer*.

[14] Mego M, Mani SA, Lee B-N (2012). Expression of epithelial-mesenchymal transition-inducing transcription factors in primary breast cancer: the effect of neoadjuvant therapy. *International Journal of Cancer*.

[15] Pecot CV, Bischoff FZ, Mayer JA (2011). A novel platform for detection of CK^+^ and CK^−^ CTCs. *Cancer Discovery*.

[16] Barrière G, Riouallon A, Renaudie J (2012). Mesenchymal and stemness circulating tumor cells in early breast cancer diagnosis. *BMC Cancer*.

[17] Ross JS, Slodkowska EA (2009). Circulating and disseminated tumor cells in the management of breast cancer. *American Journal of Clinical Pathology*.

[18] Zhao S, Liu Y, Zhang Q (2011). The prognostic role of circulating tumor cells (CTCs) detected by RT-PCR in breast cancer: a meta-analysis of published literature. *Breast Cancer Research and Treatment*.

[19] Liu MC, Shields PG, Warren RD (2009). Circulating tumor cells: a useful predictor of treatment efficacy in metastatic breast cancer. *Journal of Clinical Oncology*.

[20] Mego M, Giorgi UD, Dawood S (2011). Characterization of metastatic breast cancer patients with nondetectable circulating tumor cells. *International Journal of Cancer*.

[21] Denlinger CE, Ikonomidis JS, Reed CE, Spinale FG (2010). Epithelial to mesenchymal transition: the doorway to metastasis in human lung cancers. *Journal of Thoracic and Cardiovascular Surgery*.

[22] Vazquez-Martin A, Oliveras-Ferraros C, Cufí S, Del Barco S, Martin-Castilloand B, Menendez JA (2010). Metformin regulates breast cancer stem cell ontogeny by transcriptional regulation of the epithelial-mesenchymal transition (EMT) status. *Cell Cycle*.

[23] Wicha MS, Hayes DF (2011). Circulating tumor cells: not all detected cells are bad and not all bad cells are detected. *Journal of Clinical Oncology*.

[24] Tveito S, Andersen K, Kåresen R, Fodstad Ø (2011). Analysis of EpCAM positive cells isolated from sentinel lymph nodes of breast cancer patients identifies subpopulations of cells with distinct transcription profiles. *Breast Cancer Research*.

[25] Sieuwerts AM, Kraan J, Bolt J (2009). Anti-epithelial cell adhesion molecule antibodies and the detection of circulating normal-like breast tumor cells. *Journal of the National Cancer Institute*.

[26] Al-Hajj M, Wicha MS, Benito-Hernandez A, Morrison SJ, Clarke MF (2003). Prospective identification of tumorigenic breast cancer cells. *Proceedings of the National Academy of Sciences of the United States of America*.

[27] Meyer MJ, Fleming JM, Ali MA, Pesesky MW, Ginsburg E, Vonderhaar BK (2009). Dynamic regulation of CD24 and the invasive, CD44^pos^CD24^neg^ phenotype in breast cancer cell lines. *Breast Cancer Research*.

[28] Charafe-Jauffret E, Ginestier C, Iovino F (2009). Breast cancer cell lines contain functional cancer stem sells with metastatic capacity and a distinct molecular signature. *Cancer Research*.

[29] Micalizzi DS, Farabaugh SM, Ford HL (2010). Epithelial-mesenchymal transition in cancer: parallels between normal development and tumor progression. *Journal of Mammary Gland Biology and Neoplasia*.

[30] Wendt MK, Allington TM, Schiemann WP (2009). Mechanisms of the epithelial-mesenchymal transition by TGF-*β*. *Future Oncology*.

[31] Mamuya FA, Duncan MK (2012). *α*V integrins and TGF-*β* induced EMT; a circle of regulation. *Journal of Cellular and Molecular Medicine*.

[32] Creighton CJ, Chang JC, Rosen JM (2010). Epithelial-mesenchymal transition (EMT) in tumor-initiating cells and its clinical implications in breast cancer. *Journal of Mammary Gland Biology and Neoplasia*.

[33] Howard S, Deroo T, Fujita Y, Itasaki N (2011). A positive role of cadherin in wnt/*β*-catenin signalling during epithelial-mesenchymal transition. *PLoS ONE*.

[34] Takebe N, Warren RQ, Ivy SP (2011). Breast cancer growth and metastasis: interplay between cancer stem cells, embryonic signaling pathways and epithelial-to-mesenchymal transition. *Breast Cancer Research*.

[35] Katoh M (2011). Network of WNT and other regulatory signaling cascades in pluripotent stem cells and cancer stem cells. *Current Pharmaceutical Biotechnology*.

[36] De Herreros AG, Peiró S, Nassour M, Savagner P (2010). Snail family regulation and epithelial mesenchymal transitions in breast cancer progression. *Journal of Mammary Gland Biology and Neoplasia*.

[37] Schmalhofer O, Brabletz S, Brabletz T (2009). E-cadherin, *β*-catenin, and ZEB1 in malignant progression of cancer. *Cancer and Metastasis Reviews*.

[38] Cantrell DA (2001). Phosphoinositide 3-kinase signalling pathways. *Journal of Cell Science*.

[39] Chau NM, Ashcroft M (2004). Akt2: a role in breast cancer metastasis. *Breast Cancer Research*.

[40] Cheng GZ, Park S, Shu S (2008). Advances of AKT pathway in human oncogenesis and as a target for anti-cancer drug discovery. *Current Cancer Drug Targets*.

[41] Dowling RJO, Goodwin PJ, Stambolic V (2011). Understanding the benefit of metformin use in cancer treatment. *BMC Medicine*.

[42] Yang MH, Hsu DSS, Wang HW (2010). Bmi1 is essential in Twist1-induced epithelial-mesenchymal transition. *Nature Cell Biology*.

[43] Mongroo PS, Rustgi AK (2010). The role of the miR-200 family in epithelial-mesenchymal transition. *Cancer Biology and Therapy*.

[44] Xiong M, Jiang L, Zhou Y (2012). The miR-200 family regulates TGF-*β*1-induced renal tubular epithelial to mesenchymal transition through smad pathway by targeting ZEB1 and ZEB2 expression. *American Journal of Physiology*.

[45] Oliveras-Ferraros C, Cufí S, Vazquez-Martin A (2011). Micro(mi)RNA expression profile of breast cancer epithelial cells treated with the anti-diabetic drug metformin: induction of the tumor suppressor miRNA let-7a and suppression of the TGF*β*-induced oncomiR miRNA-181a. *Cell Cycle*.

[46] Persson J, Bäckström M, Johansson H, Jirström K, Hansson GC, Ohlin M (2009). Molecular evolution of specific human antibody against muc1 mucin results in improved recognition of the antigen on tumor cells. *Tumor Biology*.

[47] Hamid SSA, Cheah SH (2011). Generation and characterization of a high-affinity monoclonal antibody for MUC1 measurement in breast cancer. *Hybridoma*.

[48] Deguchi T, Tanemura M, Miyoshi E (2010). Increased immunogenicity of tumor-associated antigen, mucin 1, engineered to express *α*-Gal epitopes: a novel approach to immunotherapy in pancreatic cancer. *Cancer Research*.

[49] Tarp MA, Sørensen AL, Mandel U (2007). Identification of a novel cancer-specific immunodominant glycopeptide epitope in the MUC1 tandem repeat. *Glycobiology*.

[50] Medoro G, Gross S, Manaresi N (2011). Use of the DEPArray platform to detect, isolate, and molecularly characterize pure tumor cells from peripheral blood samples enriched using the CellSearch system. *Journal of Clinical Oncology*.

[51] Ståhlberg A, Kubista M, Åman P (2011). Single-cell gene-expression profiling and its potential diagnostic applications. *Expert Review of Molecular Diagnostics*.

